# Green-Synthesized Silver Nanoparticles (AgNPs) Enhance In Vitro Multiplication and Rooting of Strawberries (*Fragaria* × *ananassa* Duchesne)

**DOI:** 10.3390/biotech14020045

**Published:** 2025-06-06

**Authors:** José Luis Aguirre-Noyola, Marco A. Ramírez-Mosqueda, Jorge David Cadena-Zamudio, José Humberto Caamal-Velázquez, Esmeralda J. Cruz-Gutiérrez, Alma Armenta-Medina

**Affiliations:** 1 Centro Nacional de Recursos Genéticos-INIFAP, Boulevard de la Biodiversidad 400, Rancho las Cruces 47600, Tepatitlán de Morelos, Jalisco, Mexico; jaguirrenoyola@outlook.com (J.L.A.-N.); cadena.jorge@inifap.gob.mx (J.D.C.-Z.); cruz.esmeralda@inifap.gob.mx (E.J.C.-G.); armentam.alma@inifap.gob.mx (A.A.-M.); 2 Colegio de Postgraduados Campus Campeche, Haltunchén—Edzná km 17.5, Sihochac 24450, Champotón, Campeche, Mexico; hcaamal@colpos.mx

**Keywords:** in vitro culture, nano-gardening, nanobiotechnology, nanomaterials

## Abstract

Nanobiotechnology applications in plant tissue culture have improved the development and physiology of explants, resulting in plants with high genetic homogeneity and phytosanitary quality. Silver nanoparticles (AgNPs) are well-known for their microbicidal properties, but their biochemical effects on plants require further exploration. In this work, green-synthesized AgNPs were evaluated in strawberry in vitro culture, photosynthetic pigment production, and acclimatization. AgNPs produced by *Lysinibacillus fusiformis* were characterized. Strawberry explants were grown in vitro on MS medium with 0, 100, 200, and 300 mg L^−1^ AgNPs at 24 ± 2 °C and a photoperiod of 16:8 h light/dark. Shoot height and number, number of leaves, number of roots, and root length were evaluated, and chlorophyll (a, b, and total) was quantified. Rooted shoots were acclimatized ex vitro on substrates containing 0 and 200 mg L^−1^ AgNPs. The results showed that low AgNPs concentrations had a positive impact on shoot multiplication, development, and rooting, but at higher concentrations, the effects decayed. However, chlorophyll production improved with increasing AgNP concentration. Shoots treated with AgNPs showed higher ex vitro survival. Our study has direct implications for the profitability and sustainability of commercial strawberry production.

## 1. Introduction

Nanobiotechnology, defined as the design, characterization, production, and biological application of nanomaterials (NMs), has emerged in recent decades as a cross-cutting discipline with innovative approaches in multiple sectors of industry and research [1,2]. NMs exhibit unique physical and chemical characteristics, ranging from very tiny size (1–100 nm) to special optical, electronic, mechanical, and quantum properties that are not expressed in bulk materials [3,4]. This is why nanobiotechnology has enabled the development of more efficient tools for crop improvement, phytosanitary control, and smart fertilization, opening new opportunities for more sustainable, precise, and resilient agriculture in the face of climate change and the limitations of conventional systems [5,6].

In this context, high-value crops, such as strawberries (*Fragaria × ananassa* Duchesne), become ideal candidates for the application of these nanotechnologies, since their fruits are highly valued due to their fragrance, unique flavor, and nutritional content, especially conferred by their flavonoids, anthocyanins, minerals, vitamins and organic acids [7,8]. According to the FAOSTAT, strawberry production in 2023 reached 10.5 million metric tons, of which China contributed almost 40%, followed by the United States, Egypt, Turkey, and Mexico [9]. Despite this, the growing demand is not fully met, making it necessary to implement effective and affordable protocols to mass-producing plants. Conventional strawberry propagation has been carried out through runners (stolons) due to the low cost of the method; the drawback is that few propagules are obtained, and these are susceptible to fungal infections [10].

Plant tissue culture (PTC) or in vitro culture is gaining relevance as an efficient option to meet the challenges of strawberry cultivation because it increases yield, reduces production costs, and ensures plant quality and safety [11,12]. Individuals obtained by micropropagation are more genetically homogeneous, which ensures stable phenological and productive behavior during field cultivation [13]. With the incorporation of NMs into PTC techniques, “nano-gardening” has emerged as a novel approach that aims to optimize the development and multiplication of explants by adding NMs with bioactive properties under in vitro conditions [14].

Silver nanoparticles (AgNPs) are one of the most widely used NMs due to their low cost, high surface reactivity, and colloidal stability [15]. They also exhibit a high versatility of synthesis, which allows their production through green synthesis methods. This approach is more environmentally friendly as it produces less chemical waste and uses microorganisms or plants as catalytic agents and nucleation sites [16]. Despite the benefits of green-synthesized AgNPs, most studies have been limited to assessing their antimicrobial and antioxidant properties [17]. Although their effects on plant growth have been documented [18], little is known about the molecular mechanisms and biochemical/physiological responses that may be involved. Describing the behavior and effects on plants associated with the concentrations, sizes, and shapes of AgNPs is relevant to optimizing nano-gardening in mass plantlet production. Therefore, in this work, we evaluated the effect of the concentration of green-synthesized AgNPs on strawberry multiplication and rooting under in vitro conditions, as well as chlorophyll production and further ex vitro acclimatization.

## 2. Materials and Methods

### 2.1. Green Synthesis of Silver Nanoparticles

Silver nanoparticles (AgNPs) were produced from a green synthesis reaction mediated by *Lysinibacillus fusiformis*, following the protocol previously described by [19]. The dried and purified AgNPs were dispersed in mQ water and the concentration in solution was determined with the EPA Method 3050B using inductively coupled plasma atomic emission spectroscopy (ICP-AES) [20]. Green synthesis testing was carried out with Fourier transform infrared spectroscopy (FTIR) using the parameters described in detail by [21]. AgNPs were analyzed with X-ray diffraction (XRD) and Scherrer’s Equation (1) was used to estimate the size of submicrometer crystallites:

D = Kλ/βcosθ,(1)

where D is the nanocrystal size; K is a dimensionless shape factor, with a value close to unit; λ is the radiation wavelength in nanometers (λ = 0.15406 nm for Cu Kα radiation); β is the full width at half maximum of the peak expressed in radians; and θ is the Bragg angle [22].

### 2.2. Plant Material

Shoots of a local strawberry variety (*Fragaria × ananassa* Duchesne) obtained from apical meristems was used as explants for in vitro and acclimatization phases. The experiments were performed at the Centro Nacional del Recursos Genéticos del Instituto Nacional de Investigaciones Forestales Agrícolas y Pecuarias (Tepatitlán de Morelos, Jalisco, Mexico).

### 2.3. AgNPs-Assisted In Vitro Culture of Strawberries

Culture flasks were prepared with MS medium [23] containing 30 g L^−1^ sucrose and 0, 100, 200, or 300 mg L^−1^ AgNPs. The pH was adjusted to 5.8 ± 0.1 and 1.7 g L^−1^ of Phytagel^TM^ was added as a gelling agent. Four disinfected explants were placed in each flask with sterile forceps and then incubated in an environmental chamber at a temperature of 24 ± 2 °C under artificial illumination (50 µmol m^−2^ s^−1^) with a photoperiod 16 h light and 8 h dark. Five replicates per treatment were performed. After 45 days of cultivation, the percentage of contamination, number, and height of shoots, as well as the number and length of roots were evaluated.

### 2.4. Quantification of Photosynthetic Pigments

The photosynthetic pigment content of plants from each treatment was determined by the method of Harbone [24] with some modifications. Two hundred and fifty milligrams of plant tissue (leaves) were cut and macerated in a mortar with a pestle in 2.5 mL of acetone (80% in water). The mixture was transferred to a 15 mL tube and was stored at −20 °C, covered from light. The mixture was ground again and filtered with cellulose filter paper (4 μm). The filtered solution was made up to 6.25 mL with acetone (80% in water) and analyzed in a spectrophotometer at 645 and 663 nm. The determinations were performed in triplicate per treatment. The concentration (mg g^−1^ FW) of chlorophyll types was estimated using Equations (2)–(4) according to [25]:

(2) $$\mathrm{Chl}\;a=\left(\left(12.70\times A₆₆₃-2.69\times A₆₄₅\right)\times V\right)\;/\;\left(1000\times \;W\right)$$

(3) $$\mathrm{Chl}\;b\;=\left(\left(22.90\times A₆₄₅-4.68\times A₆₆₃\right)\times V\right)\;/\;\left(1000\times W\right)$$

(4) $$\mathrm{Total}\;\mathrm{chlorophyll}\;\mathrm{content}\;=\left(\left(20.20\times A₆₄₅+8.02\times A₆₆₃\right)\times V\right)\;/\;\left(1000\times W\right)$$

where A is the absorbance (OD) at a specific wavelength, V is the total volume of acetone extract (mL), W is the sample fresh weight (g), and 1000 is the conversion factor.

### 2.5. Acclimatization Procedure

Shoots with optimal root development and a height of 3 cm were selected and rinsed with sterile water to remove excess MS medium. Subsequently, they were planted in a sterile substrate of peat moss + agrolite (1:1 *v*/*v*) in plastic containers (15 × 15 × 8 cm). Two treatments were included: 0 and 200 mg L^−1^ of AgNPs in quintuplicate. The AgNP concentration was chosen because it induced the best results during the in vitro phase. The plantlets were fertilized with a 3 g L^−1^ solution of BayFolan^®^ Forte (Bayer de México, S.A. de C.V, Ciudad de México, México) and were maintained under greenhouse conditions with 50% shade, relative humidity between 90 ± 5%, and temperature between 30 ± 5 °C. Every 3 days the substrate was irrigated with 5 mL of water using a Truper FDO-2 domestic sprayer (Grupo Truper, S.A. de C.V, Estado de México, México). To facilitate adaptation to environmental conditions, perforations were made in the base of the containers to allow for water drainage, and the lid was gradually opened to control the system’s humidity. After six weeks of cultivation, the % survival was evaluated with Equation (5) according to [26]:

Survival rate (%) = [𝑁𝑢𝑚𝑏𝑒𝑟 𝑜𝑓 survived 𝑝𝑙𝑎𝑛𝑡𝑠/𝑇𝑜𝑡𝑎𝑙 𝑛𝑢𝑚𝑏𝑒𝑟 𝑜𝑓 𝑝𝑙𝑎𝑛𝑡𝑠] × 100(5)

### 2.6. Statistical Analysis

A Completely Randomized Design (CRD) was used in all experiments. The data obtained were statistically processed with Prism v10.4.2. An analysis of variance (ANOVA) followed by Tukey’s test (*p* ≤ 0.05) was performed to compare the means of morphophysiological parameters at different concentrations of AgNPs under in vitro conditions. An unpaired *t*-student test was performed to compare the % survival to acclimatization with and without AgNPs.

## 3. Results

### 3.1. Characterization of Green-Synthesized AgNPs

**Figure 1:**
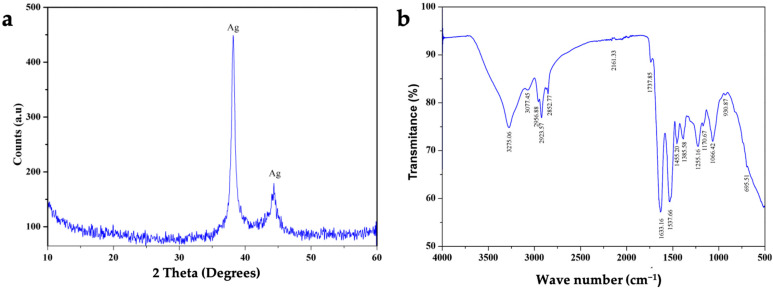
Composition and structure analysis of silver nanoparticles (AgNPs) produced by *L. fusiformis.* (**a**) X-ray diffraction (XRD) profile and (**b**) infrared spectroscopy profile of green-synthesized AgNPs. The XRD spectrum shows two primary peaks at 2θ≈38° and 2θ≈45°, which typically correspond to silver signals. The FTIR spectrum reveals bands indicative of O-H stretching (around 3400 cm^−1^), C-H stretching (around 2900 cm^−1^), and a possible C=O group (between 1500 and 1600 cm^−1^).

The AgNPs produced by *L. fusiformis* had a crystallite size of about 10–11 nm, which is typical of biogenic nanoparticles with a high degree of crystallinity. The XRD spectra revealed two primary peaks at 2θ≈38° and 2θ≈45° (Figure 1a). The first peak is intense and narrow, indicating high crystallinity, which typically corresponds to the crystalline (111) plane of metallic silver (Ag^0^). The other peak was less intense but indicative of a silver signal within the (200) crystalline plane. These results confirm that cubic crystal structure AgNPs have formed, as is common in green synthesis. The absence of additional intense peaks in the diffractogram indicates the high crystalline purity of the silver metal phase. FTIR analysis was performed to identify the functional groups responsible for reducing metal ions into nanoparticles. The FTIR spectra (Figure 1b) showed bands indicative of O-H stretching (around 3400 cm^−1^), C-H stretching (around 2900 cm^−1^), and a possible C=O group (between 1500 and 1600 cm^−1^).

Together, this information confirms that the AgNPs were the result of green synthesis by microbial action. Finally, multi-elemental quantification with ICP-AES revealed the aqueous suspension of AgNPs to be of high purity (99.2% Ag), with trace amounts of Mg (0.6%) and Fe (0.2%).

### 3.2. Effect of AgNPs on the In Vitro Propagation of Strawberries

**Figure 2:**
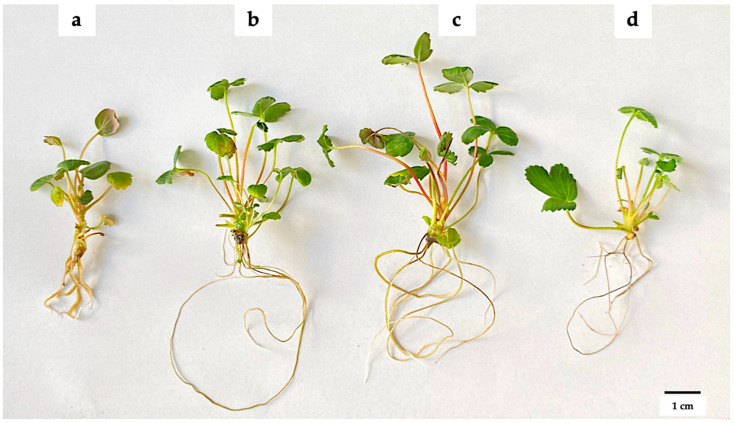
Effect of green-synthesized AgNPs on the in vitro propagation of strawberries. (**a**) 0 mg L^−1^ AgNPs, (**b**) 100 mg L^−1^ AgNPs, (**c**) 200 mg L^−1^ AgNPs, and (**d**) 300 mg L^−1^ AgNPs. The explants were grown on MS medium supplemented with AgNPs for 45 days at 24 ± 2 °C and a photoperiod of 16:8 h light/dark.

After four weeks of in vitro cultivation, it was observed that the addition of AgNPs had a concentration-dependent effect on plant growth (Figure 2). No microbial growth or endophyte presence was observed in any of the propagated tissues. Table 1 shows that adding 100 or 200 mg L^−1^ AgNPs increased the number of shoots by up to twofold (2.83–3.08 shoots), compared to the control without AgNPs (1.50 shoots). This effect declined at a concentration of 300 mg L^−1^ AgNPs, where an average of two shoots were produced. Although there were no significant differences, the presence of AgNPs tended to increase shoot lengths. Conversely, a higher number of leaves was observed in plants without AgNP addition (10.34 leaves), and no significant differences were observed among the three concentrations evaluated when AgNPs were added to the MS medium (5.75–7.63 leaves).

### 3.3. AgNPs-Induced Shoot Rooting

**Figure 3:**
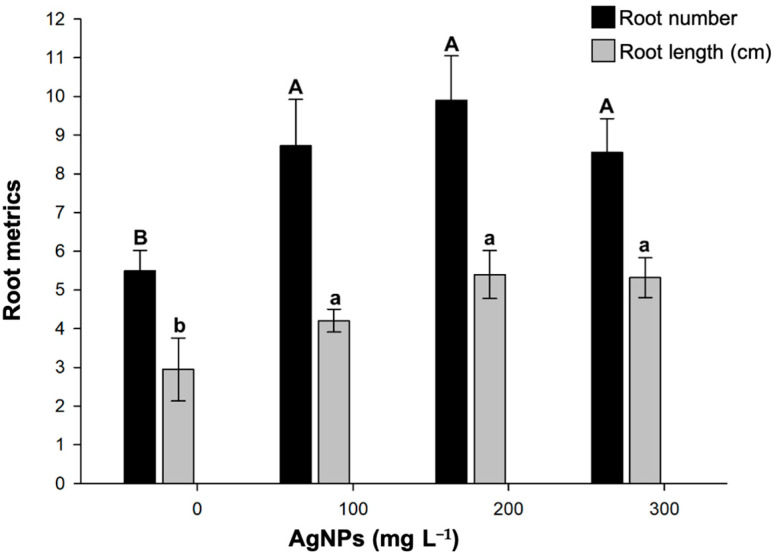
Impact of green-synthesized AgNPs on in vitro rooting of strawberries. Different upper- or lower-case letters between AgNP concentrations indicate statistically significant differences (*p* ≤ 0.05). Mean ± standard error is shown.

**Figure 4:**
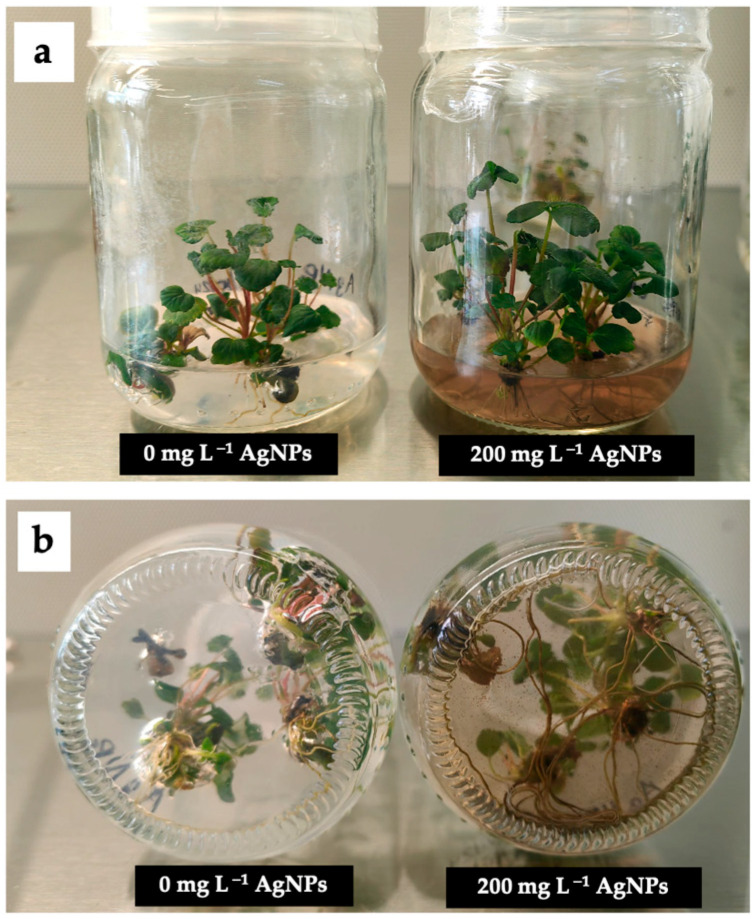
Comparison of in vitro multiplication and rooting of strawberries during nano-gardening with or without green-synthesized AgNPs. (**a**) Root development of strawberry shoots and (**b**) accumulation of AgNPs (dark aggregates) in roots at 200 mg L^−1^.

Another parameter that showed a favorable response to the incorporation of AgNPs was root system development (Figure 3). All treatments with AgNPs induced the formation of a higher number of roots (8.55–9.90), almost twice the number formed without treatment (5.50). Regarding root length, the 200 mg L^−1^ concentration of AgNPs produced roots measuring 5.40 cm. This was followed by the 300 mg L^−1^ concentration with roots measuring 5.31 cm, and finally, the 100 mg L^−1^ concentration, with roots measuring 4.20 cm. All of these lengths were significantly longer than those without Ag treatment. An interesting finding was the presence of brown aggregates in the root tissue (see Figure 4). This suggests that AgNPs accumulated in strawberry roots. AgNPs exhibit surface plasmon resonance (SPR); therefore, when exposed to light, the AgNP aggregates appear dark. Further studies are needed to investigate silver accumulation in strawberries.

### 3.4. Chlorophyll Production of Strawberry Leaves in Response to AgNPs

**Figure 5:**
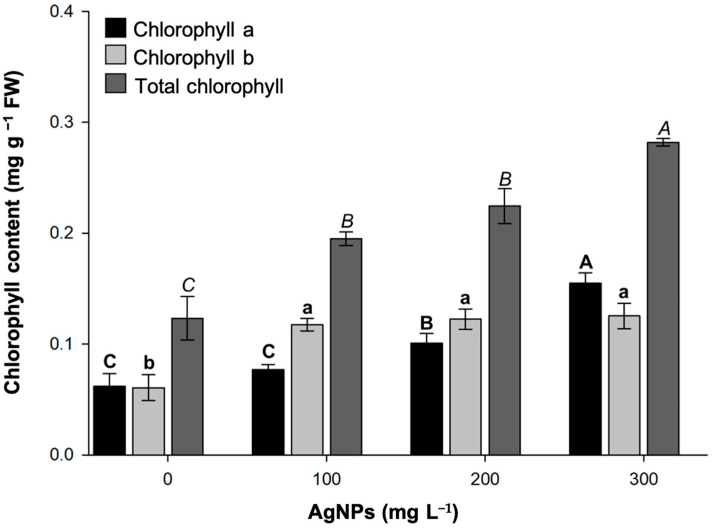
Chlorophyll content in strawberry leaves grown in vitro at different concentrations of green-synthesized AgNPs. Mean ± standard error is shown. Columns with different letters are statistically different (*p* ≤ 0.05).

The photosynthetic pigments of strawberry plants were quantified to study their physiological response to AgNPs (Figure 5). Overall, the addition of AgNPs was found to enhance chlorophyll production. Increases in chlorophyll concentration were observed only in the 200 and 300 mg L^−1^ treatments. The presence of 100 mg L^−1^ AgNPs was insufficient to induce changes in chlorophyll compared to the untreated plants. Conversely, there were no significant differences in chlorophyll b content among the 100, 200, and 300 L^−1^ AgNP concentrations; however, all were higher than the values determined in the control treatment. Finally, total chlorophyll gradually increased, reaching a maximum peak at 300 mg L^−1^ AgNPs, with approximately 0.3 mg g^−1^ FW. This value was twice that quantified in leaves in AgNP-free shoots.

### 3.5. Acclimatization to Greenhouse Conditions

**Figure 6:**
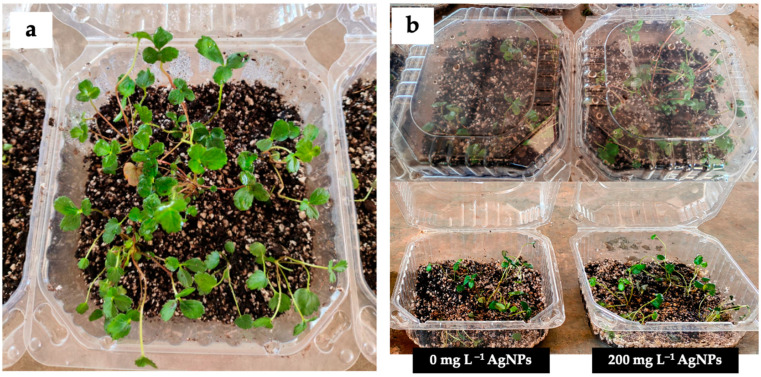
Acclimatization process of strawberries. (**a**) Plants growing in peat moss and agrolite substrate (1:1 *v*/*v*) and (**b**) comparison of acclimatization with and without AgNPs after 6 weeks at greenhouse conditions.

A critical step in the commercialization of plantlets is the adaptation of the micropropagated material to greenhouse or field conditions. In this regard, rooted strawberry shoots, both in the presence and absence of AgNPs, were transferred to a sterile substrate of peat moss and agrolite (1:1 *v*/*v*). The concentration of 200 mg L^−1^ was selected for this experiment, as it induced a more developed root system. In agreement with the in vitro results, the plants exposed to AgNPs showed a better adaptation to the substrate, which resulted in individuals with greater vigor and size (Figure 6a). After 6 weeks, the survival rate of plants with AgNPs reached 90%, which was significantly higher than the 50% observed in the treatment without AgNPs (Figure 6b). Interestingly, plants grown with AgNPs showed better performance in preliminary experiments of 50% irrigation reduction.

## 4. Discussion

Nanobiotechnology is gradually expanding its applications in agriculture, facilitating the transition to more profitable and sustainable production models [27]. Nanomaterials (NMs) can be used at various stages of food production, including seed nano-priming, in vitro cultivation, field nanofertilization, and post-harvest handling to extend the shelf life of fruits and vegetables [28]. Additionally, due to concerns about the environmental impact of NMs, green synthesis methods derived from microbial metabolism are being implemented more frequently [29]. The use of silver as a plant tissue disinfectant agent prior to micropropagation protocols has been explored [30]. Our results revealed that green-synthesized AgNPs also act as nanobiostimulants. Nanoparticles can induce molecular responses in plant cells by generating reactive oxygen species (ROS) at controlled levels, which activates stress- and growth-related signaling pathways. This stimulation can modulate the expression of genes associated with cell division, phytoalexin synthesis, and antioxidant mechanisms [31,32].

In terms of strawberry shoot production and height, the results observed with 100 and 200 mg L^−1^ of AgNPs are similar to those induced by plant growth regulators (PGRs) such as meta-Topolin (mT, 0.5 and 1.0 mg L^−1^) and 6-benzylaminopurine (BAP, 0.5, and 1.5 mg L^−1^) but lower than those recorded with zeatin (ZT, 1.0 mg L^−1^) [12,33]. The beneficial results of applying chemically synthesized AgNPs to strawberry micropropagation were also described by [34]. At a concentration of 0.2 mg L^−1^, increases in shoot number, height, and dry weight were observed. However, AgNPs at concentrations of 0.25–2.0 mg L^−1^ were unable to induce significant rooting [34]. Typically, phytohormones are added after the multiplication phase of plant tissue to induce rooting. However, this was not necessary with the green-synthesized AgNPs. Our study highlighted the ability of green-synthesized AgNPs to enhance root development, which was much higher than that caused by mT, BAP, and gibberellic acid (GA3) [12,33]. The effect promoted by AgNPs is comparable to that induced by traditional rooting agents such as indole-3-butyric acid (IBA), as reported in commercial strawberry cultivars such as ‘Sweet Charlie’ and ‘Winter Dawn’ [35].

Nonetheless, the positive effects associated with nano-gardening with AgNPs were attenuated at a concentration of 300 mg L^−1^. This behavior is suggestive of the hormesis phenomenon, i.e., low doses of NPs can elicit adaptive responses in plants, while high doses can cause nanotoxicity. In other studies, using chemically synthesized AgNPs, NMs have been associated with increased plant regeneration and accumulation of secondary metabolites, as reported in wheat (*Triticum aestivum*) [36], potato (*Solanum tuberosum*) [37], tomato (*Solanum lycopersicum*) [38], and date palm (*Phoenix dactylifera*) [39]. In medicinal plants such as passion flower (*Passiflora* spp.) [40], chirata (*Swertia chirayita*) [41], caralluma (*Caralluma tuberculata*) [42], and Vietnamese ginseng (*Panax vietnamensis*) [43], similar physiological consequences have been reported, where the use of NPs has favored growth, regeneration, and production of secondary metabolites. Contrasted with this, other research describes impairment of photosynthetic systems, oxidative stress, and cell death associated with AgNP toxicity [44,45,46]. Again, this clearly reflects that the morphological and biochemical impacts of AgNPs vary among plant species and that there are differential responses determined by the NM concentration.

The mechanisms involved in the interaction between plants and AgNPs have not yet been fully revealed. The entry of AgNPs is primarily regulated by their size, shape, and chemical properties, as well as the porosity of plant tissues [47]. In this study, AgNPs were added to the culture medium and were likely adsorbed by the root surface, passing through the fibrous matrix of the cell wall [46]. Once inside the roots, it has been proposed that AgNPs can be mobilized by symplastic (intracellular space) or apoplastic (extracellular space) transport and then use the xylem to reach the plant’s aerial parts [48]. Although we did not evaluate the AgNP distribution in strawberry tissues, we noted the accumulation of silver in their roots. First, this finding could explain why the hormetic effects were more evident at the root level. Second, it suggests that AgNPs are not easily translocated to the aerial parts. Silver accumulation in the roots may occur through adsorption to the cell wall, chelation (phytochelatins and metallothioneins), and compartmentalization in vacuoles [49]. Root accumulation is advantageous for consumers because it reduces the risk of fruit containing traces of AgNPs. Likewise, the retention of AgNPs in the roots protects photosynthetic tissues from damage caused by non-essential elements, such as silver. Accordingly, the concentration of chlorophyll in leaves did not decrease with increasing concentrations of AgNPs.

Another benefit of nanomaterials is their ability to orchestrate responses to abiotic stress through the activation of antioxidant systems, the accumulation of osmoprotectants, and the regulation of phytohormones, such as abscisic acid [28]. In this sense, 200 mg L^−1^ of AgNPs promoted a better adaptive response in strawberry plants during acclimatization experiments, reaching 90% survival. A similar survival rate (86.67–93.33%) has been reported by [34] applying 0.5 mg L^−1^ of AgNPs. Together, these results demonstrate the ability of these silver-based nanomaterials to increase tolerance to environmental changes. Effects caused by AgNPs are comparable with the survival rates achieved in strawberry plants treated with the hormones naphthaleneacetic acid (NAA, 1μM) or indole-3-butyric acid (IBA, 2 μM) in a greenhouse setting [50].

During this stage, the plants must adjust to reduced relative humidity, increased light intensity, a change in the nutrient source, and the presence of microorganisms and possible phytopathogens in the environment for the first time [51,52]. An adequate acclimatization protocol ensures a greater number of viable plants for transplanting or sale. It optimizes resource use, improves plant material uniformity, and guarantees better agronomic performance in the field. Ultimately, this translates to greater efficiency in the strawberry propagation system and a more competitive, sustainable commercial offering.

## 5. Conclusions

The biotechnological potential of green-synthesized AgNPs as nanobiostimulants for strawberry in vitro propagation was demonstrated. AgNPs at a concentration of 100–300 mg L^−1^ induced differential responses in shoot morpho-physiological development and rooting. These responses were most evident in the number and length of shoots, compared to treatments without AgNPs. It appears that the positive effects are mediated by hormetic responses, such as growth stimulation at low doses and harmful effects at high concentrations. Chlorophyll production improved with increasing AgNP concentration. From an applied perspective, the use of AgNPs during acclimation improved plant adaptation and survival under ex vitro conditions by up to 90%. This has direct implications for the profitability and sustainability of commercial strawberry production by reducing economic losses and improving the quality of propagated material. However, further characterization of the mechanisms of action and distribution of AgNPs in different plant compartments is necessary, as are long-term studies on their ecological and human health impacts. This will help ensure the safe and efficient use of nanomaterials, particularly those obtained through green synthesis, in modern agriculture.

## Figures and Tables

**Table 1:** Variable responses to AgNPs addition during in vitro propagation of strawberries.

AgNPs (mg L^−1^)	Number of Shoots	Shoot Length (cm)	Number of Leaves
0	1.50 ± 0.23 b	4.44 ± 0.35 a	10.34 ± 1.45 a
100	3.08 ± 0.64 a	5.05 ± 0.20 a	6.78 ± 0.90 b
200	2.83 ± 0.27 a	4.10 ± 0.37 a	5.75 ± 0.27 b
300	2.00 ± 0.24 b	4.78 ± 0.50 a	7.63 ± 0.57 b

a,b = Values within a column followed by different letters are significantly different according to statistical analysis (*p* < 0.05). Data are shown as mean ± standard error (SE).

## Data Availability

The original contributions presented in this study are included in the article. Further inquiries can be directed to the corresponding author(s).
